# Chloroform/Methanol Protein Extraction and In-solution Trypsin Digestion Protocol for Bottom-up Proteomics Analysis

**DOI:** 10.21769/BioProtoc.5055

**Published:** 2024-08-20

**Authors:** Tess Puopolo, Navindra P. Seeram, Chang Liu

**Affiliations:** 1 Department of Biomedical and Pharmaceutical Sciences, College of Pharmacy, University of Rhode Island, Kingston, RI, USA; 2 Proteomics Facility, College of Pharmacy, University of Rhode Island, Kingston, RI, USA

**Keywords:** Proteomics, Sample preparation, In-solution digestion, Protein extraction, Chloroform/methanol

## Abstract

Bottom-up proteomics utilizes sample preparation techniques to enzymatically digest proteins, thereby generating identifiable and quantifiable peptides. Proteomics integrates with other omics methodologies, such as genomics and transcriptomics, to elucidate biomarkers associated with diseases and responses to drug or biologics treatment. The methodologies employed for preparing proteomic samples for mass spectrometry analysis exhibit variability across several factors, including the composition of lysis buffer detergents, homogenization techniques, protein extraction and precipitation methodologies, alkylation strategies, and the selection of digestion enzymes. The general workflow for bottom-up proteomics consists of sample preparation, mass spectrometric data acquisition (LC-MS/MS analysis), and subsequent downstream data analysis including protein quantification and differential expression analysis. Sample preparation poses a persistent challenge due to issues such as low reproducibility and inherent procedure complexities. Herein, we have developed a validated chloroform/methanol sample preparation protocol to obtain reproducible peptide mixtures from both rodent tissue and human cell line samples for bottom-up proteomics analysis. The protocol we established may facilitate the standardization of bottom-up proteomics workflows, thereby enhancing the acquisition of reliable biologically and/or clinically relevant proteomic data.

Key features

• Tissue/cell pellet sample preparation for bottom-up proteomics.

• Chloroform/methanol protein extraction from murine tissue samples.

• In-solution trypsin digestion proteomics workflow.

## Graphical overview


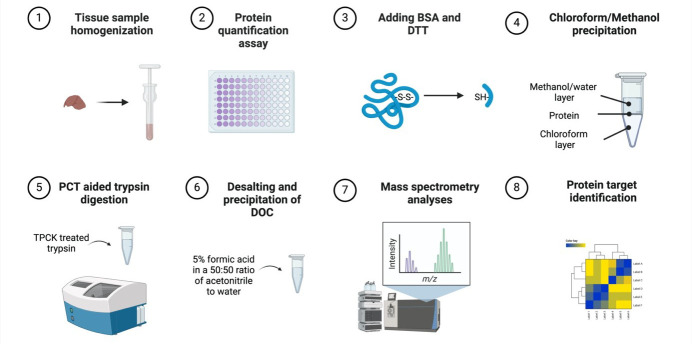


**Murine tissue sample chloroform/methanol protein extraction workflow for bottom-up proteomics analyses**

## Background

Sample preparation is a critical component of bottom-up proteomics analyses, yet it is often hindered by time constraints, performer errors, and intensive processes. Further, there remains a scientific need to develop reproducible, precise, and successful sample preparation protocols to advance proteomic discovery [1]. There are numerous sample preparation methods due to differences in sample type, protein heterogeneity, and physicochemical properties, which creates difficulty in standardizing workflows [2,3]. In-solution digestion (ISD) primarily employs varied buffers such as denaturants and detergents to denature protein samples followed by protein precipitation with organic solvents, such as chloroform/methanol, alcohol, or acetone [2]. A recent novel ISD protocol, sample preparation by easy extraction and digestion (SPEED), denatures proteins without the use of denaturants and detergents but rather through the use of trifluoracetic acid [2]. In-gel digestion (IGD), in contrast to ISD, involves the separation of proteins by size via gel electrophoresis, which has the capacity to reduce contaminants [4]. IGD is often coupled with SDS-PAGE, which utilizes the strong detergent SDS [5,6]. SDS may decrease the efficacy of proteases, such as trypsin, as well as reduce the signal/noise ratio, thereby limiting proteome coverage [5]. Therefore, it is advantageous to avoid the use of SDS in sample digestion steps or buffer solutions to prevent adverse effects and SDS removal steps. Similarly, another sample preparation method popularly employed includes device-based approaches such as filter-aided sample preparation (FASP) where SDS is also added to protein samples [7]. Samples are then filtered, and the protein is digested to elute peptides. However, FASP sample preparation protocols are time-consuming and have resulted in considerable variability, leading to the development of other methods [7]. Recently, the suspension trapping (S-Trap) method has garnered attention due to its protein extraction method via denatured size, which has improved time constraints and complexities in sample preparation [8]. However, the S-Trap method typically uses 5% SDS as a lysis buffer, and the inherent disadvantages of SDS still remain [7].

Therefore, ISD remains a promising sample preparation method compared to other approaches, yet standardization of methods for animal and cell culture samples is warranted. Herein, we developed an ISD protocol consisting of denaturation, alkylation, chloroform/methanol protein extraction, and digestion steps, as ISD methods are commonly employed in sample preparation of animal tissue samples [9]. Specifically, ISD complemented by a chloroform/methanol protein extraction method may be a reliable technique for animal and human tissue samples bottom-up proteomics as it is a fairly straightforward and fast method, which prevents protein degradation [10]. Chloroform/methanol protein extraction is a quantitative method first developed in 1984, utilizing a phase separation to precipitate proteins in solution; it has since been shown to be advantageous over acetone and ethanol for total peptides and peptides without missed cleavages [2,11]. Further, chloroform/methanol protein extraction produces a protein pellet that does not contain contaminants without reducing protein quantities [11]. In comparison to IGD, ISD is less error-prone and costly and has a higher likelihood of high peptide yield [2,4]. Further, studies have found that ISD is advantageous to identify peptides from the whole proteome, while other methods such as FASP may be better suited for specific protein recovery such as membrane proteins [12]. Our bottom-up proteomics tissue sample preparation technique using classic ISD and chloroform/methanol protein extraction methods provides a valid and reproducible workflow with protein yields suitable for in-depth, whole-proteome analyses.

## Materials and reagents

**Biological materials**

1. Frozen mouse tissue sample
*Note: This protocol has also been successfully conducted using cell lysis samples in the following manuscripts:*
  - Li et al. (2024). Anti-Ferroptotic Effect of Cannabidiol in Human Skin Keratinocytes Characterized by Data-Independent Acquisition-Based Proteomics. *J Nat Prod*. DOI: 10.1021/acs.jnatprod.3c00759. (Figures 3 & 4) [13].
  - Li et al. (2023). Cannflavins A and B with Anti-Ferroptosis, Anti-Glycation, and Antioxidant Activities Protect Human Keratinocytes in a Cell Death Model with Erastin and Reactive Carbonyl Species. *Nutrients.* DOI: 10.3390/nu15214565. (Figure 2) [14].

**Reagents**

1. Dry ice (Airgas)
2. Urea (Sigma-Aldrich, catalog number: 51457-500 mL)
3. Triethylammonium bicarbonate buffer (TEAB) (Sigma-Aldrich, catalog number: T7408-100 mL)
4. Pierce^TM^ BCA Protein Assay kit with dilution-free^TM^ BSA protein standards (Thermo Fisher Scientific, catalog number: A55865)
5. Bovine serum albumin (BSA) (Sigma-Aldrich, catalog number: A9418-5G)
6. DL-Dithioreitol (DTT) (Sigma-Aldrich, catalog number: D9779-1G)
7. Iodoacetamide (IAA) (Sigma-Aldrich, catalog number: I1149-5G)
8. Ammonium bicarbonate (Sigma-Aldrich, catalog number: A6141-25G)
9. Sodium deoxycholate (DOC) (Sigma-Aldrich, catalog number: 30970-25G)
10. Trypsin w/ CaCl_2_ (TPCK-treated), 10 pack (SCIEX, catalog number: 4445250)
11. Methanol CHROMASOLV LC-MS (Honeywell, catalog number: 34966-4L)
12. Chloroform-isoamyl alcohol mixture (Sigma-Aldrich, catalog number: 25666-100ML)
13. Milli-Q water
14. Formic acid (Sigma-Aldrich, catalog number: 695076-100ML)
15. Acetonitrile (ACN) CHROMASOLV LC-MS (Honeywell, catalog number: 34967-4X4L)

**Solutions**

1. TEAB (50 mM) and urea (8M) homogenization buffer (see Recipes)
2. 50 mM ammonium bicarbonate (pH ~8) containing 3% w/v sodium deoxycholate (DOC) (see Recipes)
3. 5% formic acid in acetonitrile and water (50:50 ratio) (see Recipes)

**Recipes**

1. **TEAB (50 mM) and urea (8M) homogenization buffer**
  **Table:** 

Reagent	Final concentration
TEAB	50 mM
Urea	8 M
2. 5**0 mM ammonium bicarbonate (pH ~8) containing 3% w/v sodium deoxycholate (DOC)**
  **Table:** 

Reagent	Final concentration
Ammonium bicarbonate	50 mM
DOC	3%
3. **5% formic acid in acetonitrile and water (50:50 ratio)**
  **Table:** 

Reagent	Final concentration
Acetonitrile	47.5%
Water	47.5%
Formic acid	5%

**Laboratory supplies**

1. Pipette Tips (TipOne 10, 200, 1,000 μL) (USA Scientific, catalog numbers: 1111-3800, 1110-1800, 1111-2821)
2. Pipettes (ErgoOne Single-Channel Pipette 2.5, 10, 200, 1,000 μL) (USA Scientific, catalog numbers: 7100-0125, 7100-0510, 7100-2200, 7110-1000)
3. Soft tissue homogenizing mix 1.4 mm ceramic (2 mL tubes) nuclease free (Omni International, catalog number: 19-627)
4. 1 mL Dounce tissue grinder (Avantor, catalog number: 357538)
5. Pour boat weigh dish 2-1/4"l × 1-3/4"w × 5/16"d, 20 mL cap (Wilkem Scientific, catalog number: 10177901)
6. 96-well tissue culture plates (Cell Treat, catalog number: 229197)
7. 96 150 μL PCT microtubes (Pressure Biosciences, Inc., catalog number: MTWS-MT-RK)
8. 150 μL PCT microcaps (Pressure Biosciences, Inc., catalog number: MTWS-MC150-RK)
9. Pipet tip gel loading .57 mm O.D. 200 μL round non-sterile (Wilkem Scientific, catalog number: LABB13790)
10. Microcentrifuge tube 0.5 mL non-sterile (Cell Treat, Wilkem Scientific, catalog number: 72316004)
11. Microcentrifuge tube 1.5 mL non-sterile (Cell Treat, Wilkem Scientific, catalog number: 229441)
12. PK100 amber glass certified S/T vial (Sigma-Aldrich, catalog number: 29386-U)
13. Advantage 150 μL volume, 5 × 30 mm bottom spring glass inserts (Analytical Sales and Services Inc., catalog number: 20501)

## Equipment

1. Bead Ruptor elite bead mill homogenizer (Omni International, model: 19-042E)
2. Microplate reader SpectraMax M2 (Molecular Devices, model: MDM2)
3. Pressure cycling technologies Barocycler (Pressure Biosciences Inc., model: NEP2320)
4. Circulating heater water bath (Thermo Fisher Scientific, model: CH 100)
5. Microcap tool Series 3 (Pressure Biosciences Inc., model: MTWS-CR-03)
6. Microtube adapter kit (Pressure Biosciences Inc., catalog number: MTTB-KEXT)
7. Centrifuge 5810 R (Eppendorf, model: 5811F)
8. Reciprocal shaking water bath (Precision Scientific, model: 66800)
9. Fisher vortex Genie 2TM (Fisher Scientific, catalog number: 12-812)

## Software and datasets

1. Prism v10.0.3 (GraphPad, 07/05/2024)

## Procedure

- **Homogenate preparation**
  1. Prepare homogenization buffer (see Recipes).
    - Add TEAB (50 mM) to urea (8 M).
  2. Place the microcentrifuge tube with tissue sample on dry ice.
  3. Place the Dounce tissue grinder glass homogenizer on dry ice.
  4. Weigh 50 mg of mouse organ tissue with a weigh boat and place into the Dounce tissue grinder.
  5. Add ~1 mL of homogenization buffer (50 mg/mL concentration) to the Dounce tissue grinder and grind the tissue sample until homogenized on dry ice.
*Note: The protein concentration and total volume may be optimized to suit the user’s specific experimental needs. For example, if using nanoflow LC systems, a lower protein concentration may be suitable. Further, large proteomic studies where many samples need to be processed for proteomics analysis may utilize smaller lysate volumes to accelerate overall procedure time.*
  6. Transfer tissue lysis into an Omni bead homogenizer tube.
  7. Homogenize tissue in an Omni bead homogenizer at 5 m/s for 30s.
  8. Centrifuge samples at 14,000× *g* for 10 min at 4 °C.
  9. Transfer the supernatant into new microcentrifuge tubes and place them on ice.
- **Protein quantification assay**
  1. Conduct BCA assay for protein quantification.
    - Use a standard curve (125–2,000 μg/mL) and multiple sample dilutions (e.g., 10-, 20-, 40-fold).
    - In a 96-well plate, add 25 μL of either standard or sample and then 200 μL of working reagent (prepared in a 50:1 ratio of reagent A to reagent B).
    - Cover the plate with aluminum foil and incubate at 37 °C for 30 min.
    - Read absorbance with the microplate reader at 562 nm.
    - In GraphPad Prism, interpolate sample protein concentrations based on a linear regression of the standard curve.
    - Prepare samples at 2,500 μg/mL (100 μL) and place them on ice for immediate use.
*Note: Samples can be frozen at -20 °C for next-day use or at -80 °C long term and then thawed at 4 °C prior to use.*
- **Adding BSA (optional) and DTT**
  1. Set the shaking water bath to 34 °C and centrifuge to 10 °C.
  2. Prepare BSA at 0.2 mg/mL in milliQ water and spike samples with 10 μL.
  3. Prepare DTT at 100 mM in milliQ water, add 25 µL to each tube, and vortex.
*Caution: Wear protective equipment when handling DTT as it is harmful if swallowed, inhaled, or with skin contact.*
  4. Denature the proteins at 34 °C in the shaking water bath for 30 min (100 rpm).
*Note: Ensure that the temperature does not exceed 35* °C. *Once urea goes over this temperature, it will break down histidine bonds and form a gelatin-like substance.*
*Note: While a short denaturation time and the use of ammonium buffer in this protocol may decrease the chances of carbamylation developing during protein reduction with heat, users may optimize the reduction step by reducing for a longer time period at room temperature, if desired.*
- **Chloroform/methanol precipitation**
  1. Place water, methanol, and chloroform on ice.
  2. Prepare iodoacetamide (IAA) (200 mM) in milliQ water and add 25 μL to each sample.
*Caution: Wear protective equipment and work under a vented fume hood when using IAA, as it is an acute toxin and harmful if swallowed, inhaled, or with skin contact.*
*Note: IAA is light-sensitive.*
  3. Place samples at room temperature (~20 °C) in the dark for 30 min for alkylation.
  4. Concentrate and precipitate samples with the sequential addition (1:2:1 ratio) of ice-cold water (160 µL), methanol (320 μL), and chloroform (160 μL) and vortex after the addition of all solvents.
*Caution: Wear protective equipment and work under a vented fume hood when using chloroform and/or methanol, as it is harmful if swallowed, inhaled, or with skin contact.*
  5. Centrifuge samples at 10,000× g for 5 min at 10 .
  6. Remove the chloroform layer from samples (bottom layer) using a gel tip (Figure 1).
*Note: Use precise care not to disrupt the pellet.*
*Critical: Removing the chloroform layer first will prevent the loss of protein sample as the protein pellet will transfer to the side of the tube.*
    **Figure 1.**
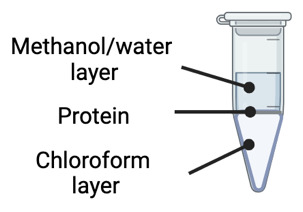
Chloroform-methanol precipitation involves a phase separation identified by an immiscible methanol/water layer on top, a protein layer in the middle, and a chloroform layer on the bottom.
  7. Remove the methanol/water layer from samples (top layer) using a gel tip.
*Note: Use precise care not to disrupt the pellet.*
  8. Add 200 μL of ice-cold methanol to the samples to wash them, and then remove it using a gel tip.
*Note: Use precise care not to disrupt the pellet. The methanol wash will remove any additional organic solvents present.*
  9. Resuspend pellet in 130 μL of 50 mM ammonium bicarbonate (pH ~8) containing 3% w/v DOC (see Recipes). Scrape walls and pipette up and down.
*Note: Samples can be stored at -80 °C if necessary, and then thawed at 4 °C prior to use.*
- **Pressure cycling technology (PCT)-aided trypsin digestion**
  1. Turn on the Barocycler and the air compressor and set the Barocycler water bath to 35–37 .
  2. Add 500 μL of milliQ water to the lyophilized TPCK-treated trypsin for a 1 mg/mL concentration and vortex.
*Note: Resuspended trypsin can be stored at -20 for future use.*
  3. Add 10 μL of trypsin (10 μg) to each sample (250 μg of protein) (at a w/w ratio of 1:25 trypsin:protein) and vortex at a medium speed (setting 4 on a scale of 0–8) for 3~5 s.
  4. Transfer 138 μL of each sample to individually labeled microtubes using gel tips.
*Note: Use care when transferring the sample into the microtubes and aim to prevent bubbles.*
  5. Cap microtubes with the microcap tool.
  6. Place microtubes into the Barocycler cartridge (Figure 2).
  7. Place the Barocycler cartridge into the PCT Barocycler instrument.
  8. Press *PRECHARGE* three times to prime the lines.
  9. Run the Barocycler for protein digestion for 75 cycles, 60 s per pressure cycle (50 s high pressure, 10 s ambient pressure), with a pressure around 25.5 psi (Figure 3).
  10. After completion of the Barocycler run, remove the Barocycler cartridge with the magnetic rod.
  11. Remove caps from microtubes with the microcap tool.
  12. Add 10 μL of trypsin to each sample (ratio of 1:25 w/w trypsin:protein) using a gel tip.
  13. Repeat PCT digestion steps as described in steps E5–E8.
  14. After completion of the Barocycler run, remove the Barocycler cartridge with the magnetic rod.
  15. Remove microtubes from the cartridge, remove caps with the microcap tool, and place the PCT tubes in the microtube rack.
*Note: PCT enhances trypsin digestion by promoting efficient and rapid protein breakdown through mechanical disruption, which accelerates the enzymatic process. Moreover, its controlled pressure cycles minimize sample variability, ensuring more reliable digestion results. In the absence of PCT, users can conduct the traditional trypsin digestion protocol, as recommended by the manufacturers, typically involving incubation with trypsin for 4–16 h at 37 °C.*
    **Figure 2.**
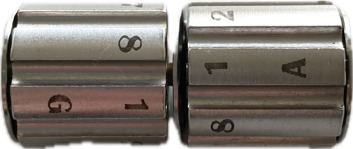
Microtube cartridges where up to 16 samples can be run on the pressure cycling technology (PCT) Barocycler for trypsin digestion.
    **Figure 3.**
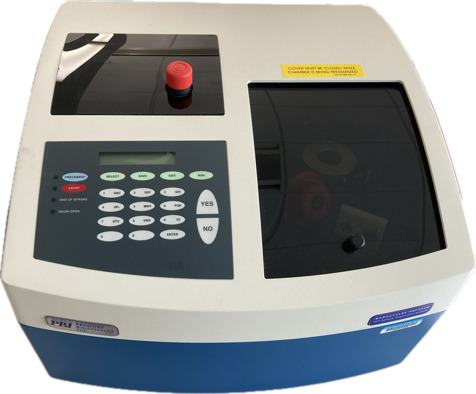
Pressure cycling technology (PCT) Barocycler instrumentation utilized for trypsin digestion of samples. The cycling methods can be programmed prior to use.
- **Desalting and precipitation of DOC**
  1. Prepare 5% formic acid in a 50:50 ratio of ACN to water and add 15 μL to 0.5 mL microcentrifuge tubes (see Recipes).
*Caution: Wear protective equipment and work under a vented fume hood, as formic acid and/or acetonitrile are harmful via all routes of exposure and flammable.*
  2. Add 135 μL of the respective samples using a gel tip to 0.5 mL microcentrifuge tubes containing 15 μL of 5% formic acid in a 50:50 ratio of ACN to water and vortex to desalt and precipitate the DOC.
*Note: A white precipitate will immediately form.*
  3. Centrifuge samples at 10,000× *g* for 5 min at 10 °C to remove precipitated DOC.
  4. Transfer sample supernatants into new 0.5 mL microcentrifuge tubes and centrifuge samples at 10,000× *g* for 5 min at 10 .
  5. Transfer 100 μL of supernatant into an amber glass vial containing a bottom spring glass insert.
  6. Samples are ready to be run for LC-MS/MS proteomics analyses.
*Note: Samples can also be stored in microcentrifuge tubes, frozen at -80 °C, thawed at 4 , and pipetted into HLPC inserts/glass vials on the day of the LC-MS/MS run.*

## Validation of protocol

This protocol or parts of it has been used and validated in the following research articles:

- Puopolo et al. (2024). Exploring immunoregulatory properties of a phenolic-enriched maple syrup extract through integrated proteomics and in vitro assays. *Food & Function* (Figures 2 and 3) [15].
- Puopolo et al. (2023). Uncovering the anti-inflammatory mechanisms of phenolic-enriched maple syrup extract in lipopolysaccharide-induced peritonitis in mice: insights from data-independent acquisition proteomics analysis. *Food & Function.* (Figures 4 and 6) [16].

## General notes and troubleshooting

**General notes**

1. Avoid multiple freeze/thaw cycles to ensure protein is not degraded.
2. This protocol is also applicable to cell lysate samples and has been validated in the following manuscripts:
  - Li et al. (2024). Anti-Ferroptotic Effect of Cannabidiol in Human Skin Keratinocytes Characterized by Data-Independent Acquisition-Based Proteomics. *J Nat Prod.* (Figures 3 and 4).
  - Li et al. (2023). Cannflavins A and B with Anti-Ferroptosis, Anti-Glycation, and Antioxidant Activities Protect Human Keratinocytes in a Cell Death Model with Erastin and Reactive Carbonyl Species. *Nutrients.* (Figure 2).
3. This protocol is also applicable to other animal tissue samples (such as rat brain) with minor sample preparation modifications and has been validated in the following manuscripts:
  - Schrader et al. (2024). Longitudinal markers of cerebral amyloid angiopathy and related inflammation in rTg-DI rats. *Sci Rep.* DOI: 10.1038/s41598-024-59013-7. (Figures 3–6) [17].
  - Schrader et al. (2021). Distinct brain regional proteome changes in the rTg‐DI rat model of cerebral amyloid angiopathy. *Journal of Neurochemistry*. DOI: 10.1111/jnc.15463. (Figures 1 and 2) [18].
4. Use extreme care not to disrupt the protein pellet during the removal of solvents or wash steps.
5. Gel tips should be used to ensure the protein pellet is not disrupted and when adding the samples to and from the PCT microtubes.
6. After sample preparation, samples can be run with any mass spectrometry and analysis method of the user’s choice.

**Troubleshooting**

Problem 1: Protein yield is low.

Possible cause 1: The sample underwent multiple freeze/thaw cycles.

Solution 1: Aim to avoid unnecessary freeze/thaw cycles as this can degrade protein, leading to lower identifiable proteins.

Possible cause 2: Incompatibility of the homogenization buffer with the sample.

Solution 2: Switching the homogenization buffer to a more suitable buffer, such as RIPA buffer, is effective for challenging proteins like nuclear or mitochondrial proteins. For example, 1 mL of RIPA buffer can replace the same volume of the current homogenization buffer (urea and TEAB) during initial homogenate preparation.
